# The extraordinary enigma of ordinary tickle behavior: Why gargalesis still puzzles neuroscience

**DOI:** 10.1126/sciadv.adt0350

**Published:** 2025-05-23

**Authors:** Konstantina Kilteni

**Affiliations:** ^1^ Donders Institute for Brain, Cognition and Behaviour, Radboud University, Nijmegen, Netherlands.; ^2^ Department of Neuroscience, Karolinska Institute, Stockholm, Sweden.

## Abstract

Gargalesis, or tickle, is one of the most trivial yet enigmatic human behaviors. We do not know how a touch becomes ticklish or why we respond to other people’s tickles but not our own. No theory satisfactorily explains why touch on some body areas feels more ticklish than on others or why some people are highly sensitive while others remain unresponsive. Gargalesis is likely the earliest trigger for laughter in life, but it is unclear whether we laugh because we enjoy it. Socrates, Aristotle, Bacon, Galileo, Descartes, and Darwin theorized about tickling, but after two millennia of intense philosophical interest, experimentation remains scarce. This review argues that gargalesis is an exhilarating scientific puzzle with far-reaching implications for developmental, sensorimotor, social, affective, clinical, and evolutionary neuroscience. We reflect on the challenges in defining and eliciting ticklish sensations in the lab and unraveling their neural mechanism, discuss five classic yet unanswered questions about tickle, and suggest directions for future research.

## Teaser

Tickle sensation remains one of the biggest unresolved mysteries in neuroscience—one that scientists are only beginning to unravel.

## INTRODUCTION

Gargalesis, commonly known as tickle, is a very familiar sensation that most of us have experienced at least once in life. Whether actively tickling our babies, family, friends, partners, or pets, or being on the receiving end of a tickle attack, humans undoubtedly engage in tickling behaviors. However, despite its triviality, the scientific understanding of gargalesis is extremely poor. Today, we do not know why certain areas of the body are more ticklish than others and why some people enjoy being tickled, while others dislike it but still burst into laughter. We have also not fully understood why we cannot tickle ourselves and why some people are very ticklish, while others are not responsive at all. Furthermore, the primary function of tickling in humans, as well as in other species, remains a big enigma.

Are these questions new, and is that why we do not have any scientific answers yet? Definitely not! Inquiries about the epistemological role of gargalesis have persisted throughout human history, from Ancient Greece to the Renaissance and beyond (*1*). Socrates (in Plato’s “Philebus”), Aristotle (in “Parts of Animals”), Desiderius Erasmus (in “Adagia”), Francis Bacon (in “Sylva Sylvarum”), Galileo Galilei (in “Il Saggiatore”), René Descartes (in “Treatise on Man” and “The Passions of the Soul”), and Charles Darwin (in “The Expression of the Emotions in Man and Animals”) all theorized about different aspects of gargalesis including its nature and underlying mechanism.

**Fig. 1.**
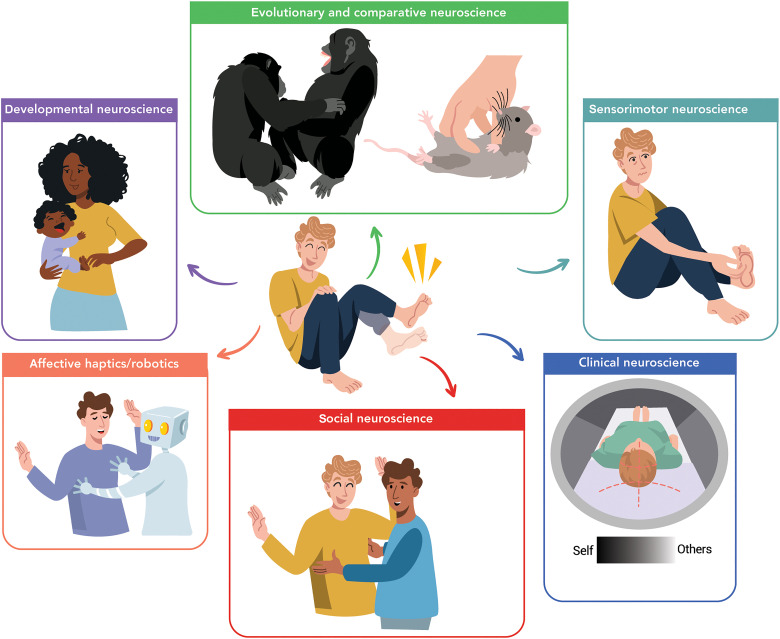
Why we should care about studying tickle sensations. The study of human tickle sensations offers profound insights for neuroscience, beyond what one might initially think. Developmental neuroscience: Responses to tickling emerge within the first year of life (*2*) and vary depending on the development stage (*3*, *4*), suggesting a potential link of tickling with sensorimotor and social development. Evolutionary and comparative neuroscience: Responses to ticklish stimuli are not exclusive to humans; they have also been observed in great apes (*6*), indicating an evolutionary basis for this sensory experience. Sensorimotor neuroscience: Tickle provides a fascinating model for understanding how the brain processes similar somatosensory input in markedly different ways depending on its origin. For example, sensations feel significantly more ticklish when induced by someone else rather than by ourselves (*15*), highlighting the brain’s predictive mechanisms and its ability to differentiate self-generated actions from external stimuli (*17*). Clinical neuroscience: Increased sensitivity to ticklish stimuli has been observed in patients with hallucinations and passivity experiences (*26*), and individuals with autism spectrum disorder (ASD) (*25*). This suggests that studying tickle responses can also help to identify sensory and sensorimotor processing abnormalities in these conditions. Social neuroscience: Tickle is likely the only touch that makes people laugh, and its neural mechanism should, at least partly, overlap with the mechanisms of social cognition, emotions, and affective processing. However, the extent to which laughter from tickling genuinely reflects joy remains unclear (*102*), making tickling a valuable model for studying these processes. Affective haptics and robotics: Research on tickle sensations presents considerable potential for affective technology, as exploring how tickle-like sensations can be recreated can make user interactions and human-machine interfaces more intuitive and emotionally engaging (*35*).

If these questions are not in any way novel to scholars past and present, then why have they failed to attract substantial interest from neuroscientists? Could the study of tickle sensation and behavior perhaps have too narrow implications to warrant further investigation? It is probably the exact opposite: Tickle is a unique model of the complex interplay between somatosensory perception, motor control, and affective processing with wide relevance across many branches of neuroscience (Fig. 1). First, from a developmental neuroscience perspective, gargalesis is one of the earliest triggers for laughter in life, and responses to tickling, defined as body movements and laughter, appear already within the first year of life and stabilize in later years (*2*). Tickling forms an important part of social play activities between parents and infants. Different responses to the ticklish touches of their mothers have been detected between infants (*3*) and toddlers (*4*), and changes in the frequency of their prelinguistic vocalization have been further observed depending on their development stage and neurodevelopmental condition (*5*). Second, research on tickling has implications for evolutionary and comparative neuroscience as, besides humans, chimpanzees, bonobos, gorillas, and orangutans respond to being tickled (*6*–*8*). Earlier studies have further suggested the existence of similarities in tickling vocalizations between humans and nonhuman primates (*6*, *7*, *9*). Moreover, research in isolate-housed rats suggests that 50-kHz ultrasonic vocalization patterns in response to human touch might represent laughter-like responses to playful tickling (*10*–*14*). Third, researchers in sensorimotor neuroscience investigate how the same touch might feel significantly more ticklish if applied by another person on our foot sole compared to when we apply it ourselves (*15*, *16*) and propose that this is because our brain can predict and suppress our own self-touches (*17*). Similar attenuation, or even cancelation, effects for the predictable self-generated sensations have been observed in other species and modalities (*18*–*24*). Fourth, from a clinical neuroscience perspective, earlier research showed that people with autism spectrum disorder (ASD) perceive touches as more ticklish than controls (*25*). In addition, patients with auditory hallucinations and passivity experiences (*26*) perceive their self-touches as similarly ticklish as external touches. Similarly, nonclinical individuals with high schizotypal traits (*27*, *28*) perceive their self-touches as more ticklish than those of nonclinical individuals with low schizotypal traits and as comparably ticklish as external touches*.* Fifth, tickle has important relevance also to social neuroscience. Most people hold the view that tickling requires a familiar social context (*29*–*32*), and we would not be tickled by strangers. On the other hand, some researchers argue that gargalesis elicits more reflex-like than social responses (*33*) and that we can get tickled by machines to the same extent as we get tickled by our cospecifics (*34*). Last, tickle has recently gained relevance in the field of affective haptics and robotics, particularly in the development of wearable haptics such as insoles (*35*), slippers (*36*), and tactile displays (*37*), as well as other technologies including handheld devices (*38*) and vibrotactile displays (*39*), that aim to induce and communicate emotions between users through tickling (*40*).

To sum up, gargalesis has occupied some of the brightest philosophical minds across the last two millennia of human history, and research on this subject could have broad implications for human developmental, social, clinical, affective, and evolutionary neuroscience. Then, why has empirical research on ticklishness fallen so short where philosophical deliberations flourished? Here, we synthesize the literature on gargalesis and suggest that our limited scientific understanding of tickle arises from three important challenges in (i) defining tickle sensations, (ii) experimentally inducing and quantifying them, and (iii) characterizing their neural mechanism. To overcome these obstacles, we propose targeted actions toward more rigorous and replicable experiments. Next, we delve into five classic, yet unaddressed, questions about gargalesis. For each question, we review previous works, discuss their potential limitations, and suggest alternative experimental approaches. We conclude that gargalesis perception (Box 1) is an exhilarating puzzle waiting to be solved by neuroscientists and that we are only at the very beginning of this journey.

Box 1. **Tickle/gargalesis sensation or tickle/gargalesis perception?**

The fact that not all touches feel ticklish, but rather that touch needs to have certain features and be applied to specific areas of the body, speaks in favor of gargalesis being a stream of distinct sensations conveyed by sensory nerve fibers. At the same time, the observation that the same touch can feel ticklish when administered by another person but not when applied by ourselves suggests that further processing and interpretation of these sensory signals are involved for a touch to be perceived as ticklish. Here, we will use the terms “tickle/gargalesis sensation” and “tickle/gargalesis perception” interchangeably.

## CHALLENGE 1. DEFINITION OF GARGALESIS

**Fig. 2.**
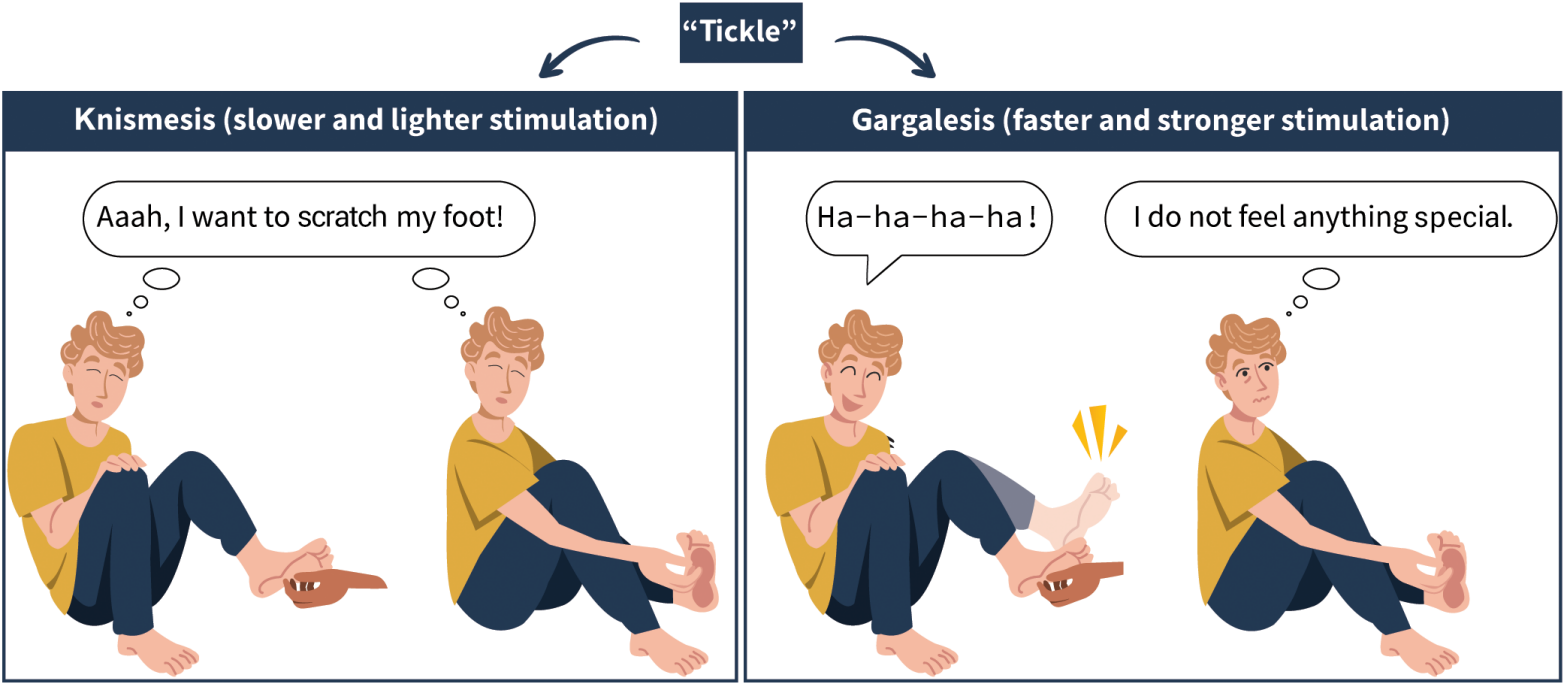
Studies refer to different sensations with the term “tickle.” **Left:** Some studies elicited and quantified mechanically induced itch sensations using slow, light feather-like tactile stimulation on all areas of the body and used the term tickle to refer to their research question. These sensations are termed knismesis and can be self-induced to a great extent. **Right:** Other studies elicited and quantified laughter-inducing tactile sensations using faster and stronger tactile stimulation on specific areas of the body, such as the torso, the armpits, and the soles of the feet. These sensations, termed gargalesis, are difficult to self-induce. The present article focuses on the latter, the gargalesis sensation, which, in contrast to knismesis, remains widely understudied.

To study a sensation in the lab, a scientist needs to know and understand its definition to successfully elicit it or experimentally manipulate it. One of the major problems with studying gargalesis is that there is no consensus within the medical and research community about what sensations are described by the word “tickle.” For some scientists of the 20th century, tickle was another term to describe itch-like sensations that are induced by applying very light mechanical stimulation to the skin [for example, using a brush, a feather, or a cotton wisp (*41*–*48*)]. This mechanically induced itch, or the “itch that moves” (*47*), can trigger a variety of responses including pulling oneself away from the stimulus or pulling the stimulus away from the body, horripilation (i.e., goosebumps) (*42*), and the urge to scratch or rub the affected skin area (*46*). Common situations in which one can experience this sensation are when wearing a woolen sweater or having an insect crawling on the arm that causes light mechanical rubbing of the skin. It is relatively easy to induce this type of sensation in yourself: Just move a feather, a brush, or your finger very lightly and slowly along your upper lip, forearm, or forehead. You will likely experience a slight discomfort or tingling sensation that outlasts the stimulation time and triggers a subsequent desire to rub the affected skin area. If you apply forceful or fast strokes, then this itchy sensation vanishes (Fig. 2, left).

Nevertheless, what we most frequently associate with the term “tickle” is a discrete and ambivalent sensation tightly coupled to a drive to convulse and laugh. This sensation arises when fast applying strong, repetitive pressure on specific areas of the body, such as the armpit, sides, or the foot soles (*2*, *34*, *49*–*52*), and typically includes body stiffening, pulling the body away from the stimulus, smiling, and laughing (*53*). This type of tickle requires a tickler and cannot be self-induced: Try to stroke your sides, waist, or armpits with your fingers. Typically, you will experience the pressure, but you will not self-induce any involuntary muscle contractions, laughter, or uneasiness (Fig. 2, right). These interactions usually involve the approach of the tickler toward the ticklee (the setup) and the actual tactile stimulation (the climax) (*4*, *5*). In some cases, the ticklee retaliates by becoming the tickler, leading to reciprocal “tickle fights,” where both individuals engage in a back-and-forth exchange (*32*, *54*).

To differentiate these two types of sensations, different terms were coined. At the end of the 19th century, the term “knismesis” from Ancient Greek κνησμός (knēsmós, “itching”) was introduced to describe itch-like sensations, and the term “gargalesis” from Ancient Greek γαργαλίζω (gargalízō, “tickle”) was used to describe laughter-inducing tickling (*53*). Alternative terms such as the “superficial tickle” and the “deep tickle” were introduced a few years later (*55*) to reflect the light feather-like touch needed for knismesis and the harder touch needed for gargalesis. Nevertheless, the prevalence of these terms in the scientific literature remains relatively limited (*56*).

In practice, experimental studies have rarely specified which sensations they aimed to elicit and study (knismesis versus gargalesis), leading to seemingly contradictory findings. For example, some studies reported that the forearms/elbows and the thighs were the most sensitive body areas to tickle (*41*, *57*), in contrast to others indicating that the elbows, palms, and legs were the least sensitive areas to tickle (*53*). However, these results can be conciliated if we consider that the former studies studied knismesis (*41*, *57*) and the latter studied gargalesis (*53*).

A similar confusion exists regarding the peripheral mechanisms of tickling as described in contemporary medical textbooks (*58*, *59*). Early animal and human physiological work suggested that ticklish sensations are detected by C low-threshold mechanoreceptive free nerve endings, conveyed through unmyelinated fibers which innervate mostly the hairy skin, following the anterolateral pathway (*45*, *47*, *48*, *60*), similar to pain, heat, cold, and itch. This pathway, however, is based on physiological findings on knismesis and not gargalesis. Gargalesis requires the application of high pressure on both hairy (e.g., armpits) and glabrous areas (e.g., foot soles) and is thus likely to activate a wide range of afferents (*61*, *62*). These include low-threshold mechanoreceptors (fast-conducting Aβ fibers and slow-conducting C fibers particularly in the hairy skin), high-threshold mechanoreceptors [including the A-fiber high-threshold mechanoreceptive nociceptors (*63*)], C-mechanosensitive nociceptors with low force activation thresholds (*61*), and even muscle afferents depending on the proximity of the muscles to the stimulation site. It is this particular intermix of gargalesis with knismesis at the physiological level that led some researchers to further assign tickling to the interoceptive senses (*64*). Originally, interoception refers to the processing of signals originating within the body and related to the state of one’s body. However, broader definitions include not only visceral (internal) sensations (*65*) but also sensations that share a similar neural pathway, as, for example, the unmyelinated nerve fiber types (*66*). Given that knismesis sensations have been linked to the activation of the unmyelinated class C–afferent fibers (*48*, *60*) among others, tickle has been included under the umbrella term of interoception although it should be emphasized that this might not apply to gargalesis.

Last but not least, it is important to distinguish between gargalesis and knismesis also when speculating the function of tickling: While knismesis can act protectively in drawing attention to the skin and urging the removal of insects, parasites, or irritants (*54*), identical to the function proposed for itch (*67*, *68*), it is the function of gargalesis that remains a big mystery (*50*).

## CHALLENGE 2. EXPERIMENTAL INDUCTION AND QUANTIFICATION OF GARGALESIS

If the definition of tickle among studies is unclear, then it should not be surprising that we currently lack established research standards. On the one hand, there are no standardized methods to experimentally elicit ticklish sensations in the lab. For example, regarding the stimulation instruments, some researchers, likely interested in knismesis, used cotton wads, brushes (*41*–*44*, *69*), feathers (*57*, *70*–*72*), soft foam (*25*, *28*, *73*–*75*), threads, pins, and hairs (*46*, *55*), while others likely interested in gargalesis involved direct skin-to-skin stimulation using the finger of the experimenter (*34*, *49*, *51*), the participant’s friend/partner (*76*, *77*), or a person within the participant’s social circle (*78*). Similarly, the body areas stimulated in experiments vary greatly; some studies likely targeting knismesis stimulated the upper limbs [palm (*25*, *28*, *73*, *74*, *79*), wrist (*37*), and forearm (*41*, *70*, *80*)], the face [forehead (*46*, *81*), cheek (*43*), and lip (*46*, *55*, *81*)], or the entire body (*57*), while other studies investigating gargalesis addressed the torso [ribs/waist (*49*, *51*, *82*)] and the foot sole (*69*, *71*). Moreover, the properties of the applied stimulation studies also greatly differ between studies. For example, some studies targeting knismesis applied very light feather-like stimulation [e.g., 0.17 N (*15*, *70*)] or instructed the experimenters to apply as light strokes as possible (*70*) on the participants’ bodies, while others oriented to gargalesis opted for more forceful stimulation [e.g., up to 15 N (*82*)]. This heterogeneity is further aggravated by limited or undetailed specifications of the stimulation parameters given to the participants. In particular, in experiments in which participants are tickled by other individuals, there are no instructions given to the ticklers about how to administer the touch, or there is no description about how this was practically accomplished (*34*, *49*, *51*). This becomes even more problematic in studies where each participant is stimulated by a different individual (*76*–*78*, *83*, *84*) (e.g., the subject’s friend).

A further problem is the lack of standardized dependent variables to quantify gargalesis. Subjective reports about how ticklish the stimulation felt are the most common way to assess tickle sensations, e.g., (*34*, *49*, *51*, *69*, *82*), but on their own, they can be subject to demand characteristics and interindividual variability, as any self-reported measures. Some research has captured behavioral reactions to ticklish touch in audio and/or video recordings, such as vocalizations (e.g., laughter), facial expressions (e.g., smiling), and body movement (e.g., movements to avoid the ticklish stimulus) (*3*–*5*, *34*, *35*, *40*, *49*, *51*, *78*, *82*). Only a small number of studies have collected physiological responses, including measurements of respiration (*40*, *78*), skin conductance response, heart rate, and skin temperature (*35*, *40*), under the premise that gargalesis changes the arousal levels and subsequently affects the activity of the autonomic nervous system. Self-reports and behavioral and physiological measures are not found to systematically correlate across studies. For example, some studies detect a relationship between subjective reports and facial/body movements (*51*), while others did not (*40*, *78*). Similarly, some experiments have detected a relationship between subjective reports and laughter (*77*, *78*, *85*), whereas others have failed to observe such a correlation (*76*) or have not found a link between tickle perception and the anticipated changes in respiration associated with laughter (*40*, *78*). The discrepancies between studies may be attributed to the different methods used as well as the differences in sample sizes and thus in the statistical power to detect responses to ticklish touches [e.g., 11 participants (*78*) and 72 participants (*51*)].

## CHALLENGE 3. NEURAL MECHANISMS OF GARGALESIS

For a neuroscientist, it is important not only to perceptually characterize triggers and responses to tickle sensations, but also to elucidate the neural mechanisms of gargalesis. Today, we know very little about it. This is due to the scarcity of neuroimaging studies on the topic, which have predominantly focused on brain activity during laughter (*76*, *77*, *83*) or tickle anticipation (*69*, *77*). At the central level, studies on the anticipation of being tickled demonstrated that tickle expectations elicited activity in the sensorimotor cortex, anterior insula, hypothalamus, nucleus accumbens, and ventral tegmental area (*69*, *77*, *85*), among other regions. Gargalesis studies showed that tickle sensations on the foot accompanied by laughter elicited activation in several sensorimotor areas, including the primary sensorimotor cortex (foot, mimic musculature, larynx, pharynx, and diaphragm areas), the supplementary motor area, and the cerebellum (*76*, *85*). In addition, tickling and laughter activated the anterior and posterior insula, hypothalamus, nucleus accumbens, ventral tegmental area, anterior cingulate cortex, and periaqueductal gray (PAG) matter (*77*, *85*). Vocal responses were found to relate to activity in the PAG, hypothalamus, insula (*76*, *83*), and midbrain tegmentum (*77*). However, the primary focus of those studies was not the neural mechanism of gargalesis, but the neural mechanism of laughter. Consequently, the conditions that were contrasted with the “ticklish” condition that involves strokes on the foot sole were not designed to provide similar but nonticklish somatosensory input, but with conditions of voluntary laughter (*76*, *83*) or with conditions where the participants received stimulation of markedly different properties (e.g., a constant pressure on the foot sole) (*77*).

## HOW TO OVERCOME THESE CHALLENGES AND MOVE FORWARD

**Fig. 3.**
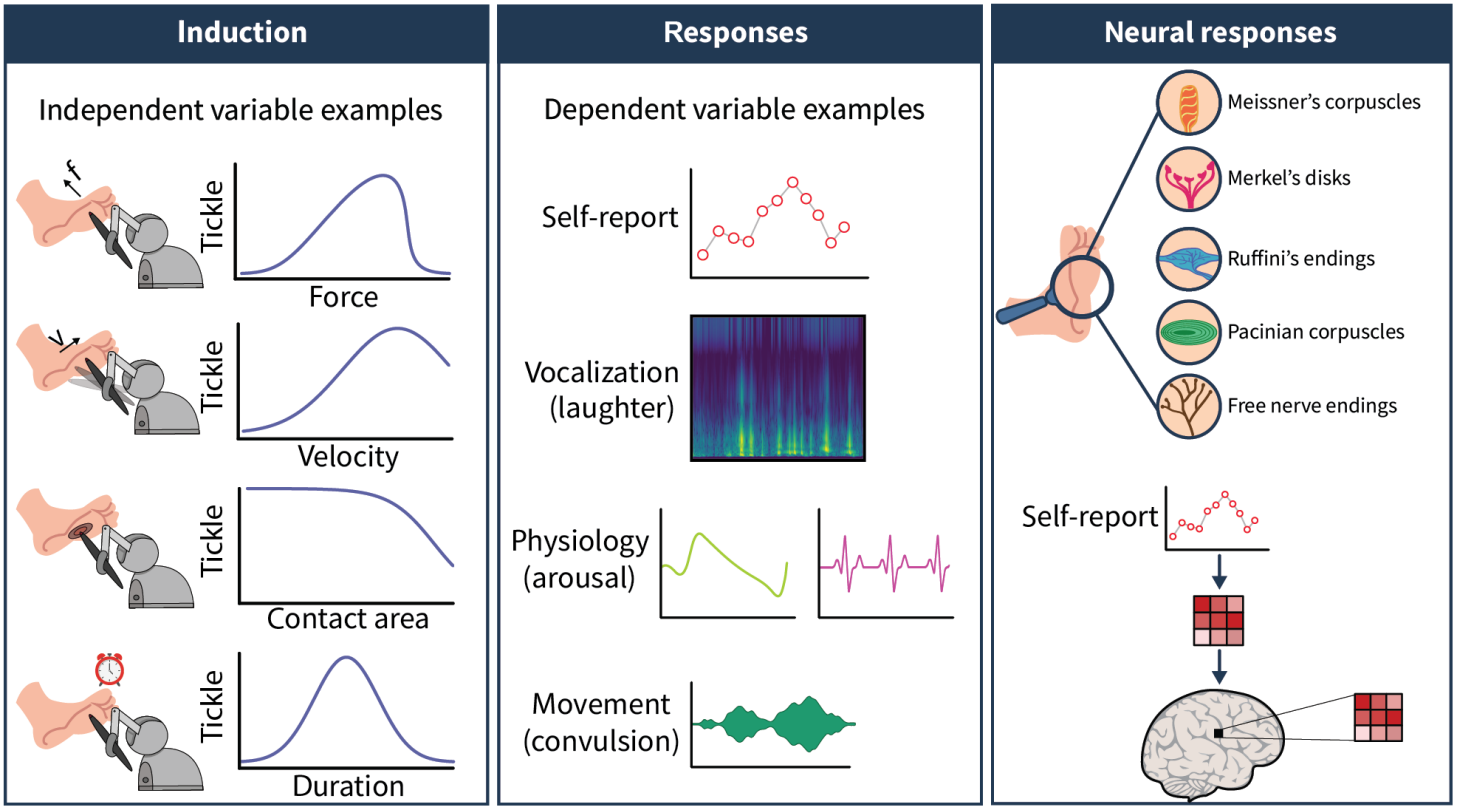
Future steps needed to understand the principles of human gargalesis sensations. **Left**: The challenges in experimentally inducing and quantifying gargalesis can only be faced with automatized methods of tactile stimulation coupled with systematic investigation of which properties a touch needs to have to feel ticklish. Future experiments need to test which stimulation parameters (e.g., force and velocity of stroke, predictability of pokes, duration of stimulation, etc.) convert touch to tickle. The graphs display hypothetical relationships between ticklishness and stimulation features. **Middle**: Successfully eliciting reliable tickle sensations in a lab will allow us to detect which measure better captures the perception and derive additional objective indices of ticklishness. For example, tickle responses typically include laughter, arousal, and convulsive movements. **Right top**: Automatized methods will further allow delineating the neural pathway of tickle sensation. At the peripheral level, there are various types of sensory receptors within the skin that convey tactile information. In glabrous skin, low-threshold mechanoreceptors include Meissner’s corpuscles, which are sensitive to light touch, slip, and texture; Merkel’s disks, which are sensitive to light touch and pressure; Ruffini endings, which are sensitive to skin stretch; Pacinian corpuscles, which are sensitive to deep pressure and vibration; and free nerve endings that respond to a wide range of sensations including touch, pressure, pain, temperature, and itch. However, because gargalesis typically requires forceful stimulation, it is expected to activate not only low-threshold but also high-threshold mechanoreceptors, nociceptors, and even muscle afferents if there are muscles close to the stimulation site. Now, we do not know which sensory receptor(s) mediate gargalesis. **Right bottom**: At the central level, neuroimaging analyses based on the participants’ reported ticklishness can relate neural activity or patterns (univariate and multivariate pattern analyses) to subjective reports of ticklishness while controlling for the physical characteristics of stimulation.

To improve the reliability and methodological rigor of tickle research, several steps should be taken. At minimum, future studies should document whether the aim is to induce gargalesis or knismesis. Second, research needs to transition from manual tactile stimulation to automated administration, using haptic devices and robots for example. This will allow for precise modulation of stimulus parameters (e.g., force of touch, velocity of stroke, area of contact, and predictability) and the optimization of stimulation protocols to reliably induce tickle sensations across individuals (Fig. 3, left). Automated procedures can promote the replicability between studies without diminishing the subjective experience. Studies using such techniques have successfully elicited tickling responses and evoked laughter responses [e.g., (*35*)], demonstrating their effectiveness. This approach aligns with similar advancements in related fields, such as affective touch and itch, where automated methods have also proven successful [e.g., see (*60*, *86*)]. Third, although ticklishness is subjective, self-reports should be systematically considered along with other variables, and methods that allow for better quantification of facial and body movements (e.g., electromyography, kinematic recordings, and video-based automated facial expression analyses) and physiological signals (e.g., electrocardiogram, electroencephalogram, skin conductance response, pupil dilation, and respiration) should become the norm (Fig. 3, middle). Statistical techniques can further explore which measures better capture the subjective perception of ticklish touch, determine how strongly physiological, kinematic, and subjective responses are related to each other, and identify common underlying factors among different measures that explain the variance in ticklishness perception.

Automated stimulation methods will also enable us to reveal the neural mechanism of gargalesis both at the peripheral and central levels (Fig. 3, right). Peripherally, in vivo electrophysiological recordings [i.e., microneurography (*87*)] require subjects to remain motionless (*88*, *89*) to prevent the displacement of the recording electrode from the specific fiber of interest. This is challenging, as gargalesis can induce body convulsion and laughter in participants. To circumvent the movement restrictions, one could alternatively test whether patients with selective loss or reduction of sensory fibers [e.g., myelinated Aβ type (*63*, *90*) or unmyelinated C type (*91*)] experience gargalesis or use software for simulating neural activations (*88*). Last, to better understand how touch is perceived as ticklish at the central level, it is important to compare stimuli that share most properties but differ in a key factor influencing perceived ticklishness. For example, strokes with identical velocity, contact area, and duration may still evoke different ticklish sensations if the applied force varies: Strokes below a certain force threshold may feel nonticklish, while those exceeding a higher force threshold may become painful. Participants’ ratings on ticklishness can further be used to model brain activity or support multivariate analyses to detect brain activity patterns that follow the participants’ ticklishness scores.

## FIVE OUTSTANDING QUESTIONS ABOUT GARGALESIS THAT WE HAVE NO ANSWER TO

**Fig. 4.**
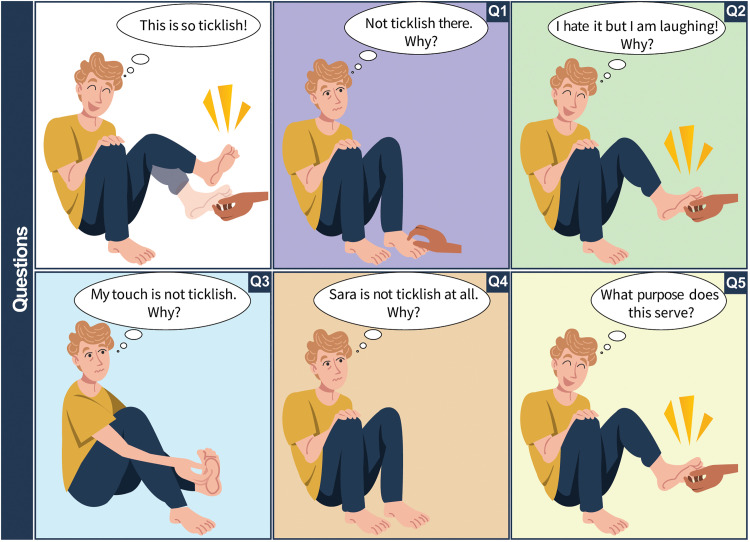
Five classic questions about gargalesis (tickle) we have no scientific answer to. Despite the intense philosophical debate over the past two millennia, gargalesis remains the most understudied somatic sensation in relation to pain, itch, and touch. Now, we do not know why our feet and armpits are reported as more ticklish than other areas of our body (Q1: spatial specificity) and why some people enjoy being tickled, while others hate it, but they still laugh (Q2: affective value). Moreover, we have not understood how our brain cancels the ticklishness of our own touches (Q3: agency) and why people vary in their sensitivity to tickle (Q4: interindividual differences). However, the biggest mystery about gargalesis remains its function in humans, as well as across other species (Q5: function).

Having outlined the challenges of gargalesis research, we now turn to five key questions that continue to intrigue both the scientific community and the general public (Fig. 4).

### Question 1. Why does touch feel more ticklish on certain areas of the body than on others?

A syllabus issued in 1896 (*53*) revealed that the foot soles and armpits are the most ticklish areas in children, and a recent survey (*92*) reported similar results in adults highlighting the feet and the armpits. Other ticklish areas include the neck, sides, stomach, and groin, among other areas (*53*, *54*, *92*). What is special about these areas? The most intuitive explanation is a physiological one according to which the most ticklish areas are those with the highest sensitivity to touch or pain. However, this account was ruled out more than a century ago (*93*) given that the armpits and foot soles are neither among the areas of highest tactile or pain acuity (*94*) nor those with the highest cutaneous mechanoreceptor density (fingertip and face) (*95*). Aristotle proposed that ticklishness depends on the skin’s delicacy; however, the skin on the foot soles and the armpits is not the thinnest (compare, for example, the eyelid) (*96*), and nonbony soft-tissue areas, such as the lips or the breasts, are not among the most ticklish areas.

Over the years, alternative nonphysiological theories have been proposed. Specifically, it was suggested that the most ticklish areas are those most vulnerable in an arm-to-arm fight (*53*). Accordingly, the armpits, sides, neck, and groin are ticklish, because an attack on these areas could cause serious injury: An assault on the armpit could damage the axillary artery, an assault on the sides could harm the abdominal cavity, an assault on the neck could injure the carotid arteries or trachea, and an assault on the inguinal region (i.e., groin) could damage the femoral vessels (*54*) (see also question 5). However, this proposal was challenged because our hands and arms are also vulnerable (e.g., an assault on our arm could injure the brachial artery) but not ticklish body areas (*50*). In addition, it was proposed that the ticklish areas might be the most erogenous (*31*), but this proposal can also be ruled out (*50*) because our armpits are not among the most sexually arousing body parts (*97*). Darwin proposed that the ticklishness of certain body areas is related to how frequently they receive external touch. He suggested that our armpits and the area between our toes are not often touched by external sources, explaining why touches to those areas are often perceived as ticklish. In addition, he theorized that ticklishness might be connected to the (a)typical patterns of touch we experience. For instance, while our foot soles receive broad surface contact when we walk and stand, this differs from the narrow finger stimulation that causes tickling. However, this explanation might be quite simplistic and not hold for other areas, as, as Darwin also noted, our buttocks and back also receive broad stimulation when we sit but are not particularly ticklish. Although Darwin’s theory warrants formal testing in future studies, it should be evident that we now have no theory that can satisfactorily explain this spatial “specificity” of gargalesis.

### Question 2. Do we enjoy being tickled, and if not, why do we laugh?

In Plato’s Philebus, Socrates describes ticklish sensations as ambivalent, with elements of both pain and pleasure. Observations in infants suggest that their experiences alternate between seemingly positive states (e.g., seeking contact) and negative states (e.g., avoiding contact) (*54*). Prolonged exposure to tickling can switch laughter to crying (*2*), and there are also reports of tickling being used as a form of punishment or torture in World War II (*98*). Experimental studies have also provided mixed results: Some indicate that overall, people enjoy being tickled (*84*) and consider it a pleasant experience (*77*), with some individuals even seeking it out or including it in their sexual behavior (*92*). However, other reports suggest that only one-third of the tested people (32% of 84 subjects) enjoy being tickled, while most either maintain a neutral impression (32% of 84 subjects) or explicitly report not enjoying it (34% of 84 subjects) (*49*). While more investigations with large sample sizes are needed to systematically assess whether tickle is positively or negatively valenced, it raises an intriguing question: Given that we laugh when tickled, does that not mean that we enjoy it?

As a social behavior, laughter can communicate distinct emotions and have different connotations, ranging from happiness and joy to even embarrassment and aggression (*99*, *100*). Gargalesis is likely the earliest trigger for laughter in life (*99*), as early as 24 weeks of life (*2*), but research indicates that tickling laughter is more dissimilar to joyful laughter than we may think. Specifically, tickling laughter has different acoustic parameters (e.g., complexity and pitch) (*101*), elicits a higher level of emotional arousal (*99*), evokes different neural activation (*102*) and brain connectivity patterns (*103*), and is perceptually distinct (*84*, *99*) from joy laughter. These findings support the view that gargalesis laughter might be a reflex-like reaction to touch, similar to “crying while cutting an onion” (*51*, *52*), and might constitute the primitive version of laughter that diversified later during evolution into socially complex emotional laughter forms (*102*).

### Question 3. Why are we unable to tickle ourselves?

Undoubtedly, gargalesis is difficult to self-induce, and an observational study in 1941 suggests that we are not able to tickle ourselves already within the first year of life (*2*). Thirty years later, in 1971, a formal experiment showed that adults feel the strokes on their foot soles to be more ticklish when applied by another person compared to themselves (*15*).

Integrating this phenomenon into his early theory of tickle perception, Darwin proposed that to feel ticklish sensations, one must be surprised about the body area that will be touched (*104*). In other words, one cannot self-tickle because he/she knows beforehand when and where the touch will be experienced. This relates to a well-established computational theory of human motor control that proposed that on each voluntary movement we perform, our brain uses information from the motor signals to predict and suppress the self-generated sensations (*17*, *105*–*108*). Consequently, when touching (or trying to tickle) our own body, the brain predicts and cancels the resulting sensations, prioritizing the processing of external stimulation such as those from our predators (*17*, *24*, *105*). In line with this, numerous experimental studies showed reduced perceived intensity and/or ticklishness (*80*, *109*–*124*) for self-generated touches compared to externally generated touches, across a wide age range (*119*, *120*) and in the vast majority of people (98%, or 315 of 322) (*120*). Similarly, neuroimaging studies revealed reduced activity in the primary (*125*) and secondary (*73*, *126*) somatosensory cortex for self-generated compared to externally generated touches [see (*18*, *21*, *127*–*130*) for analogous findings in other species]. Attenuated cerebellar responses (*73*, *126*) and increased functional cerebellar connectivity with somatosensory areas (*126*) were also observed during self-generated touches compared to external touches, in line with the view that the cerebellum is engaged in this predictive attenuation of touch (*131*–*136*).

To date, there is a general consensus that the most likely reason we cannot tickle ourselves is due to this mechanism of attenuating the perception of self-generated touches. However, all previous neuroimaging studies have either used nonticklish touches (e.g., forces and taps) (*73*, *125*, *126*, *134*, *135*, *137*) or applied light tactile stimuli on the participants’ palms to induce knismesis (*73*) rather than gargalesis. Therefore, experiments using ticklish touch, in terms of gargalesis, are needed to conclusively demonstrate that this attenuation mechanism underlies self-tickle cancelation. Moreover, while self-generated touch feels less intense than externally generated touch, it generally feels not ticklish at all rather than just less ticklish than external touch: For example, when we try to tickle our own armpits, we do not experience any ticklish sensations. This suggests that the perception of ticklishness might be more binary depending on whether the touch is self-generated or externally generated (ticklish versus nonticklish), as opposed to the perception of intensity, which can gradually vary in degree (stronger versus weaker).

### Question 4. Why are some people very ticklish while others are unresponsive?

The extent to which earlier gargalesis studies have accurately captured individual differences in perceived ticklishness is difficult to assess. This is primarily due to the variability that manual tactile stimulation inherently involves, which could contribute to perceptual variability: If every participant is touched differently, then the perceived ticklishness will also vary between individuals. In addition, we lack studies with large sample sizes to assess overall differences in reported body ticklishness across the general population. However, large individual differences in gargalesis are reasonable to expect, as an earlier study reported that when receiving the same automated tactile stimulation, one-third of the participants were nonresponders, and one-third were strong responders (*40*).

Understanding the sources of these differences is an important challenge because our tactile experiences extend beyond mere feature extraction of afferent signals produced by mechanoreceptors. Instead, they result from a unique and complex mixture of factors, ranging from trait-like genetic and physiological factors (*95*, *138*–*140*) to more transient psychological and cognitive states (*141*–*143*). At the peripheral neural level, for example, it has been proposed that people with smaller fingers have lower thresholds of tactile spatial acuity (*144*) because the density of mechanoreceptors is likely higher in smaller limbs (*145*, *146*). Besides finger size, recent results have further suggested that differences in skin stiffness affect how the skin surface deforms during contact with objects and can explain individual differences in tactile acuity (*147*). At the central neural level, it has been proposed that individuals with larger somatosensory resting state fluctuations have better tactile discrimination ability (*148*), while those with greater gray matter volume in early somatosensory areas have greater somatosensory sensitivity (*149*). Although these findings relate to tactile acuity and not to ticklishness, they illustrate that the perception of touch, and, by extension, the perception of ticklishness, can vary between individuals due to many factors.

It is also generally assumed that children are more ticklish than adults. From a developmental perspective, it has been proposed that this increased sensitivity might help children to develop their propensity for laughter, and, consequently, their sense of humor in their later life (*30*), if one accepts a link between tickle and humor (see also question 2). Moreover, being tickled might help juveniles learn how to defend their bodies in physical fights later in life (*54*) (see also question 5). Under this theory, children might have an increased “appetite for tickling” due to their need to learn tactics for self-defense. However, ticklishness differences between children and adults could also stem from an increased thrill-seeking behavior for touch in children rather than ticklishness per se (*52*). Last, social perception research suggests that certain personality dimensions, such as neuroticism or extroversion, can also influence how we process touch (*150*, *151*), and earlier research showed that participants who rated themselves as more ticklish also reported laughing or crying more easily than others (*30*).

One should also consider that ticklishness may be state dependent. Darwin suggested that one’s mind needs to be in “a pleasurable condition” to experience ticklishness. In contrast, Francis Bacon suggested that “men even in a grieved state of mind, yet cannot sometimes forbear laughing” when tickled (*52*). An earlier study aimed to test whether placing some subjects into a pleasant state of mind by watching comedy films would increase their tickle responses but found no effects. Being tickled also did not make participants laugh more while watching the comedy clips (*51*). Although these results seem to contradict the impact of mental state, future studies are needed to explore this relationship more thoroughly. In addition, longitudinal studies could provide insights into how changes in mental state over time might influence gargalesis perception.

### Question 5. Why do we experience tickle sensations?

Does tickle have a concrete function, or is it a by-product of other tactile perceptions with no evolutionary advantage or disadvantage? The function of gargalesis has been hotly debated for centuries, and the question remains to the largest degree unresolved.

Tickling has been proposed to be essential for survival, representing a form of “mock battles” that prepare juveniles for warfare by teaching them tactics and strategies for defending their bodies in real fights (*54*). According to this theory, ticklishness serves as a way to train young individuals to protect vulnerable areas of the body, such as the armpits, neck, and groin, during fight simulations. Proponents of this view argue that tickling played a crucial role for our ancestors (e.g., apes) and early humans, but as humans transitioned from physical combat to the use of tools and weapons for self-defense, learning warfare techniques through physical contact became less substantial (*54*).

According to the social account, tickling is a playful behavior that serves to foster bonds among partners, friends, and family by establishing a “tactile communication channel” between the “tickler” and the “ticklee” (*29*). It serves a similar social function for great apes as it does for humans (*29*). Furthermore, it contributes to the development of humor in infants (*30*) and transitions into sexual play in adulthood (*31*), carrying a sexual connotation (*31*, *92*, *152**, **153*). Proponents of this view argue that gargalesis requires the involvement of two people (*29*, *154*) and relies on a specific social context: Tickle sensations can only be induced by people with whom we have close social relationships (*29*), while the same touch would be perceived as unpleasant and nonticklish if applied by strangers (*54*, *104*).

Opponents of the social view emphasize that gargalesis triggers stereotypical sensorimotor “reflex-like” startle responses (*33*), including convulsive movements to evade the tickler. They argue that if gargalesis were of a social nature, then it is paradoxical that we instinctively move away from the tickling stimulus. Alternatively, gargalesis might be a stimulus-sensitive sensation where neither the social context nor the state of mind is critical (*34*). Accordingly, tickling could serve to alert and protect our bodies from potentially aversive stimuli, similar to other reflex responses.

An earlier study (*34*) aimed to contrast the social with the reflex theory of tickle by testing whether participants’ laughter responses differed between being tickled by a human and being tickled by a human while believing they were being tickled by a “machine.” The study failed to observe any differences between the two conditions, supporting the reflex view of gargalesis. However, the machine the participants believed was tickling them was designed to look similar to a human hand, thereby imposing an anthropomorphic context [see also (*82*) for hand-shaped tickle devices]. To contrast the two theories, future studies should test the strength of tickle sensations when participants experience identical tactile stimulation but in different social contexts; for example, when the tactile stimulation is delivered by a haptic machine that is thought to be controlled by another human (e.g., a friend or a stranger) or when it is preprogrammed. Moreover, one should not exclude the possibility that tickling sensations can be induced by a machine but are amplified within a rich social context.

## CONCLUDING REMARKS

The work discussed here illustrates both the challenges and expected implications of studying human tickle perception. So far, experimentation has been hindered by the lack of consensus in its definition, the absence of established experimental methods and measures, and the resulting obstacle toward understanding the neural mechanism of tickle. However, a promising message from this article is that researchers can observe the complexity of tickle perception in laboratory settings. New haptic technologies and advanced research methodologies permit future studies to go further and enrich this line of inquiry by developing experimental frameworks to investigate tickle sensation. Moving forward, it is also critical that studies acknowledge the dissociable relationship between knismesis (itch) and gargalesis (tickle).

As highlighted here, there is still so much to know about the mechanisms of tickling. First, it is fundamental for future studies to understand the physical triggers of tickle sensations: What properties, conditions, and contexts render a touch to a tickle. From a methodological and experimental design perspective, understanding these triggers is necessary for effectively addressing the questions about tickle sensations in the lab. Second, a major goal of future research will be to study the neural pathway of tickling from the periphery to the brain, as well as how the brain cancels the ticklishness of our own touches (question 3). Understanding how our nervous system cancels the ticklishness of our own touches is essential for understanding how the distinction between ourselves and others is neurally implemented. This is critical, as there is evidence that self-other distinction can be altered in certain clinical conditions, such as schizophrenia. Third, it could be that identifying the function of tickling (question 5) may ultimately depend on answering other questions first: insights from ticklish body areas (question 1), emotional responses (question 2), and individual differences (question 4) could provide evidence—either supporting or refuting—its proposed protective or social bonding function. Toward this direction, tickling is a behavior that would greatly benefit from a close partnership between human and animal experimentation. Tickling behaviors share great similarities between humans and great apes, and potentially other mammals, and animal and human researchers can work in synergy to answer the key question about the evolutionary function of tickling.

To conclude, the field will greatly benefit from a qualitative breakthrough, which can be achieved through research standardization. We are therefore optimistic that new insights into this uncharted territory of tickle research are on the horizon and that modern neuroscience will soon be able to provide explanations for what puzzled Socrates, Aristotle, Erasmus, Bacon, Galileo, Descartes, and Darwin.

## References

[1] J. Moshenska, *Feeling Pleasures: The Sense of Touch in Renaissance England* (OUP Oxford, 2014).

[2] C. Leuba, Tickling and laughter: Two genetic studies. Pedagog. Semin. J. Genet. Psychol. 58, 201–209 (1941).

[3] K. Ishijima, K. Negayama, Development of mother–infant interaction in tickling play: The relationship between infants’ ticklishness and social behaviors. Infant Behav. Dev. 49, 161–167 (2017). 28934614 10.1016/j.infbeh.2017.08.007

[4] K. Matsushima, T. Kato, An exploratory study on the association between atypical behavioral responses to tickling and autistic traits in Japanese children. Occup. Ther. Health Care 38, 666–685 (2023). 10.1080/07380577.2023.2297358 38174406

[5] H. C. Hsu, S. N. Iyer, A. Fogel, Effects of social games on infant vocalizations. J. Child Lang. 41, 130–152 (2014). 10.1017/S0305000912000608 23298621

[6] M. Davila Ross, M. J. Owren, E. Zimmermann, Reconstructing the evolution of laughter in great apes and humans. Curr. Biol. 19, 1106–1111 (2009). 19500987 10.1016/j.cub.2009.05.028

[7] M. D. Ross, M. J. Owren, E. Zimmermann, The evolution of laughter in great apes and humans. Commun. Integr. Biol. 3, 191–194 (2010). 20585520 10.4161/cib.3.2.10944 PMC2889984

[8] B. van Boekholt, R. Wilkinson, S. Pika, Bodies at play: The role of intercorporeality and bodily affordances in coordinating social play in chimpanzees in the wild. Front. Psychol. 14, 1206497 (2023). 38292528 10.3389/fpsyg.2023.1206497 PMC10826840

[9] J. Vettin, D. Todt, *Human Laughter, Social Play, and Play Vocalizations of Non-human Primates: An Evolutionary Approach* (Brill, 2005).

[10] J. Panksepp, J. Burgdorf, 50-kHz chirping (laughter?) in response to conditioned and unconditioned tickle-induced reward in rats: Effects of social housing and genetic variables. Behav. Brain Res. 111, 25–38 (2000). 10996405 10.1016/s0166-4328(00)00238-2

[11] J. Burgdorf, J. Panksepp, Tickling induces reward in adolescent rats. Physiol. Behav. 72, 167–173 (2001). 11239994 10.1016/s0031-9384(00)00411-x

[12] J. Panksepp, J. Burgdorf, “Laughing” rats and the evolutionary antecedents of human joy? Physiol. Behav. 79, 533–547 (2003). 12954448 10.1016/s0031-9384(03)00159-8

[13] N. Gloveli, J. Simonnet, W. Tang, M. Concha-Miranda, E. Maier, A. Dvorzhak, D. Schmitz, M. Brecht, Play and tickling responses map to the lateral columns of the rat periaqueductal gray. Neuron 111, 3041–3052.e7 (2023). 37516112 10.1016/j.neuron.2023.06.018 PMC10552647

[14] S. Ishiyama, M. Brecht, Neural correlates of ticklishness in the rat somatosensory cortex. Science 354, 757–760 (2016). 27846607 10.1126/science.aah5114

[15] L. Weiskrantz, L. Elliot, C. Darlington, Preliminary observations of tickling oneself. Nature 230, 598–599 (1971). 4928671 10.1038/230598a0

[16] K. Kilteni, “Methods of somatosensory attenuation,” in *Somatosensory Research Methods*, N. P. Holmes, Ed. (Springer, 2023), pp. 35–53.

[17] D. McNamee, D. M. Wolpert, Internal models in biological control. Annu. Rev. Control Robot. Auton. Syst. 2, 339–364 (2019). 31106294 10.1146/annurev-control-060117-105206 PMC6520231

[18] D. M. Schneider, J. Sundararajan, R. Mooney, A cortical filter that learns to suppress the acoustic consequences of movement. Nature 561, 391–395 (2018). 30209396 10.1038/s41586-018-0520-5 PMC6203933

[19] N. J. Audette, W. Zhou, A. La Chioma, D. M. Schneider, Precise movement-based predictions in the mouse auditory cortex. Curr. Biol. 32, 4925–4940.e6 (2022). 36283411 10.1016/j.cub.2022.09.064 PMC9691550

[20] S. Chinta, S. R. Pluta, Neural mechanisms for the localization of unexpected external motion. Nat. Commun. 14, 6112 (2023). 37777516 10.1038/s41467-023-41755-z PMC10542789

[21] A. Wallach, N. B. Sawtell, An internal model for canceling self-generated sensory input in freely behaving electric fish. Neuron 111, 2570–2582.e5 (2023). 37321221 10.1016/j.neuron.2023.05.019 PMC10524831

[22] N. B. Sawtell, Neural mechanisms for predicting the sensory consequences of behavior: Insights from electrosensory systems. Annu. Rev. Physiol. 79, 381–399 (2017). 27813831 10.1146/annurev-physiol-021115-105003

[23] J. X. Brooks, J. Carriot, K. E. Cullen, Learning to expect the unexpected: Rapid updating in primate cerebellum during voluntary self-motion. Nat. Neurosci. 18, 1310–1317 (2015). 26237366 10.1038/nn.4077 PMC6102711

[24] J. X. Brooks, K. E. Cullen, Predictive sensing: The role of motor signals in sensory processing. Biol. Psychiatry Cogn. Neurosci. Neuroimaging 4, 842–850 (2019). 31401034 10.1016/j.bpsc.2019.06.003 PMC6733654

[25] S. J. Blakemore, T. Tavassoli, S. Calò, R. M. Thomas, C. Catmur, U. Frith, P. Haggard, Tactile sensitivity in Asperger syndrome. Brain Cogn. 61, 5–13 (2006). 16500009 10.1016/j.bandc.2005.12.013

[26] S.-J. Blakemore, J. Smith, R. Steel, C. E. Johnstone, C. D. Frith, The perception of self-produced sensory stimuli in patients with auditory hallucinations and passivity experiences: Evidence for a breakdown in self-monitoring. Psychol. Med. 30, 1131–1139 (2000). 12027049 10.1017/s0033291799002676

[27] A.-L. Lemaitre, M. Luyat, G. Lafargue, Individuals with pronounced schizotypal traits are particularly successful in tickling themselves. Conscious. Cogn. 41, 64–71 (2016). 26891191 10.1016/j.concog.2016.02.005

[28] T. J. Whitford, A. M. Mitchell, D. J. Mannion, The ability to tickle oneself is associated with level of psychometric schizotypy in non-clinical individuals. Conscious. Cogn. 52, 93–103 (2017). 28500871 10.1016/j.concog.2017.04.017

[29] R. R. Provine, “Yawns, laughs, smiles, tickles, and talking: Naturalistic and laboratory studies of facial action and social communication,” in *The Psychology of Facial Expression*, J. A. Russel, J. M. Fernández-Dols, Eds. (Cambridge Univ. Press, 1997), pp. 158–175.

[30] A. J. Fridlund, J. M. Loftis, Relations between tickling and humorous laughter: Preliminary support for the Darwin-Hecker hypothesis. Biol. Psychol. 30, 141–150 (1990). 2285764 10.1016/0301-0511(90)90023-p

[31] R. R. Provine, *Curious Behavior: Yawning, Laughing, Hiccupping, and Beyond* (Harvard Univ. Press, 2012).

[32] R. R. Provine, Laughing, tickling, and the evolution of speech and self. Curr. Dir. Psychol. Sci. 13, 215–218 (2004).

[33] D. W. Black, Laughter. JAMA 252, 2995–2998 (1984). 6502861

[34] C. R. Harris, N. Christenfeld, Can a machine tickle? Psychon. Bull. Rev. 6, 504–510 (1999). 12198790 10.3758/bf03210841

[35] D. S. Elvitigala, R. Boldu, S. Nanayakkara, D. J. C. Matthies, TickleFoot: Design, development and evaluation of a novel foot-tickling mechanism that can evoke laughter. ACM Trans. Comput. Human Interact. 29, 1–23 (2022).

[36] Y. Kume, “Foot interface: Fantastic phantom slipper,” in *ACM SIGGRAPH 98 Conference Abstracts and Applications* (Association for Computing Machinery, 1998), p. 114.

[37] E. Knoop, J. Rossiter, “The tickler: A compliant wearable tactile display for stroking and tickling,” in *Conference on Human Factors in Computing Systems* (Association for Computing Machinery, 2015), vol. 18, pp. 1133–1138.

[38] M. Furukawa, H. Kajioto, S. Tachi, Shared palm for remote tickling. Int. J. of Adv. Comput. Sci. 3, 90–98 (2013).

[39] Y.-W. Park, C.-Y. Lim, T.-J. Nam, “CheekTouch: An affective interaction technique while speaking on the mobile phone,” in *Conference on Human Factors in Computing Systems* (Association for Computing Machinery, 2010), pp. 3241–3246.

[40] P. E. Fortin, J. R. Cooperstock, Laughter and tickles: Toward novel approaches for emotion and behavior elicitation. IEEE Trans. Affect. Comput. 8, 508–521 (2017).

[41] V. Ruggieri, M. Milizia, Tickle perception as micro-experience of pleasure: Its phenomenology on different areas of the body and relation to cerebral dominance. Percept. Mot. Skills 56, 903–914 (1983). 6877978 10.2466/pms.1983.56.3.903

[42] V. Ruggieri, M. Milizia, M. F. Romano, Effects of body image on tactile sensitivity to a tickle: A study of pregnancy. Percept. Mot. Skills 44, 555–563 (1979). 10.2466/pms.1979.49.2.555 514774

[43] V. Ruggieri, M. Milizia, F. Angeli, Reaction to cutaneous (tickle) and sexual pleasure by normal and dermapathic subjects. Percept. Mot. Skills 61, 903–910 (1985). 4088783 10.2466/pms.1985.61.3.903

[44] V. Ruggieri, M. Milizia, N. Sabatini, M. T. Tosi, Body perception in relation to muscular tone at rest and tactile sensitivity to tickle. Percept. Mot. Skills 56, 799–806 (1983). 6877967 10.2466/pms.1983.56.3.799

[45] Y. Zotterman, Touch, pain and tickling: An electro-physiological investigation on cutaneous sensory nerves. J. Physiol. 95, 1–28 (1939). 16995068 10.1113/jphysiol.1939.sp003707 PMC1393960

[46] D. T. Graham, H. Goodell, H. G. Wolff, Neural mechanisms involved in itch, “itchy skin,” and tickle sensations. J. Clin. Investig. 30, 37–49 (1951). 14803555 10.1172/JCI102414 PMC436225

[47] T. Mintz, Tickle--The itch that moves. A psychophysiological hypothesis. Psychosom. Med. 29, 606–611 (1967). 5582954 10.1097/00006842-196711000-00005

[48] M. Nordin, Low-threshold mechanoreceptive and nociceptive units with unmyelinated (C) fibres in the human supraorbital nerve. J. Physiol. 426, 229–240 (1990). 2231398 10.1113/jphysiol.1990.sp018135 PMC1189885

[49] C. R. Harris, N. Alvarado, Facial expressions, smile types, and self-report during humour, tickle, and pain. Cogn. Emot. 19, 655–669 (2005).

[50] C. R. Harris, “Tickling,” in *Encyclopedia of Human Behavior: Second Edition* (Academic Press, 2012) pp. 611–615.

[51] C. R. Harris, N. Christenfeld, Humour, tickle, and the Darwin-Hecker hypothesis. Cogn. Emot. 11, 103–110 (1997).

[52] C. R. Harris, The mystery of ticklish laughter. Am. Sci. 87, 344 (1999).

[53] G. S. Hall, A. Alliń, The psychology of tickling, laughing, and the comic. Am. J. Psychol. 9, 1–41 (1897).

[54] L. Robinson, The science of ticklishness. North. Am. Rev. 185, 410–419 (1907).

[55] E. A. B. Pritchard, The clinical significance of variations in tickle sensibility. Proc. R. Soc. Med. 26, 697–704 (1933). 19989246 10.1177/003591573302600607 PMC2204487

[56] A. A. Varlamov, I. V. Skorokhodov, Knismesis: The aversive facet of tickle. Curr. Opin. Behav. Sci. 43, 230–235 (2022).

[57] S. Svebak, The importance of skin area and gender in ticklishness. Scand. J. Psychol. 62, 683–688 (2021). 34152014 10.1111/sjop.12756

[58] J. E. Hall, A. Guyton, *Textbook of Medical Physiology E-Book* (Elsevier Health Sciences, 2020).

[59] R. Greger, U. Windhorst, *Comprehensive Human Physiology: From Cellular Mechanisms to Integration* (Springer, 1996).

[60] M. Fukuoka, Y. Miyachi, A. Ikoma, Mechanically evoked itch in humans. Pain 154, 897–904 (2013). 23582153 10.1016/j.pain.2013.02.021

[61] R. Ackerley, “Somatosensation and body perception: The integration of afferent signals in multisensory cognitive processes,” in *Cognitive Archaeology, Body Cognition, and the Evolution of Visuospatial Perception* (Elsevier, 2023), pp. 3–23.

[62] A. Handler, D. D. Ginty, The mechanosensory neurons of touch and their mechanisms of activation. Nat. Rev. Neurosci. 22, 521–537 (2021). 34312536 10.1038/s41583-021-00489-x PMC8485761

[63] S. S. Nagi, A. G. Marshall, A. Makdani, E. Jarocka, J. Liljencrantz, M. Ridderström, S. Shaikh, F. O’neill, D. Saade, S. Donkervoort, A. R. Foley, J. Minde, J. Cole, C. G. Bönnemann, A. T. Chesler, M. C. Bushnell, F. Mcglone, H. Olausson, An ultrafast system for signaling mechanical pain in human skin. Sci. Adv. 5, eaaw1297 (2019). 31281886 10.1126/sciadv.aaw1297 PMC6609212

[64] S. S. Khalsa, R. Adolphs, O. G. Cameron, H. D. Critchley, P. W. Davenport, J. S. Feinstein, J. D. Feusner, S. N. Garfinkel, R. D. Lane, W. E. Mehling, A. E. Meuret, C. B. Nemeroff, S. Oppenheimer, F. H. Petzschner, O. Pollatos, J. L. Rhudy, L. P. Schramm, W. K. Simmons, M. B. Stein, K. E. Stephan, O. Van den Bergh, I. Van Diest, A. von Leupoldt, M. P. Paulus, Interoception Summit 2016 participants, Interoception and mental health: A roadmap. Biol. Psychiatry Cogn. Neurosci. Neuroimaging 3, 501–513 (2018). 29884281 10.1016/j.bpsc.2017.12.004 PMC6054486

[65] A. D. Craig, How do you feel? Interoception: The sense of the physiological condition of the body. Nat. Rev. Neurosci. 3, 655–666 (2002). 12154366 10.1038/nrn894

[66] H. D. Critchley, S. N. Garfinkel, Interoception and emotion. Curr. Opin. Psychol. 17, 7–14 (2017). 28950976 10.1016/j.copsyc.2017.04.020

[67] F. Cevikbas, E. A. Lerner, Physiology and pathophysiology of itch. Am. Physiol. Soc. 100, 945–982 (2020). 10.1152/physrev.00017.2019 PMC7474262 31869278

[68] N. K. Wimalasena, G. Milner, R. Silva, C. Vuong, Z. Zhang, D. M. Bautista, C. J. Woolf, Dissecting the precise nature of itch-evoked scratching. Neuron 109, 3075–3087.e2 (2021). 34411514 10.1016/j.neuron.2021.07.020 PMC8497439

[69] K. Carlsson, P. Petrovic, S. Skare, K. M. Petersson, M. Ingvar, Tickling expectations: Neural processing in anticipation of a sensory stimulus. J. Cogn. Neurosci. 12, 691–703 (2000). 10936920 10.1162/089892900562318

[70] G. Claxton, Why can’t we tickle ourselves? Percept. Mot. Skills 41, 335–338 (1975). 1178428 10.2466/pms.1975.41.1.335

[71] A. N. Cunningham, “The effect of gender on tickle sensitivity: A test of the interpersonal theory of tickle,” thesis, Villanova University (2005).

[72] O. K. Faseyitan, “Investigation of an anomalous non-linear decline in tickle sensitivity,” thesis, Villanova University (2009).

[73] S.-J. Blakemore, D. M. Wolpert, C. D. Frith, Central cancellation of self-produced tickle sensation. Nat. Neurosci. 1, 635–640 (1998). 10196573 10.1038/2870

[74] S.-J. Blakemore, C. D. Frith, D. M. Wolpert, Spatio-temporal prediction modulates the perception of self-produced stimuli. J. Cogn. Neurosci. 11, 551–559 (1999). 10511643 10.1162/089892999563607

[75] S.-J. Blakemore, D. M. Wolpert, C. D. Frith, The cerebellum contributes to somatosensory cortical activity during self-produced tactile stimulation. Neuroimage 10, 448–459 (1999). 10493902 10.1006/nimg.1999.0478

[76] E. Wattendorf, B. Westermann, K. Fiedler, E. Kaza, M. Lotze, M. R. Celio, Exploration of the neural correlates of ticklish laughter by functional magnetic resonance imaging. Cereb. Cortex 23, 1280–1289 (2013). 22508768 10.1093/cercor/bhs094

[77] E. Wattendorf, B. Westermann, K. Fiedler, S. Ritz, A. Redmann, J. Pfannmöller, M. Lotze, M. R. Celio, Laughter is in the air: Involvement of key nodes of the emotional motor system in the anticipation of tickling. Soc. Cogn. Affect. Neurosci. 14, 837–847 (2019). 31393979 10.1093/scan/nsz056 PMC6847157

[78] S. Proelss, S. Ishiyama, E. Maier, M. Schultze-Kraft, M. Brecht, The human tickle response and mechanisms of self-tickle suppression. Philos. Trans. R. Soc. B Biol. Sci. 377, 20210185 (2022). 10.1098/rstb.2021.0185 PMC9489287 36126671

[79] M. Blagrove, S.-J. Blakemore, B. R. J. Thayer, The ability to self-tickle following rapid eye movement sleep dreaming. Conscious. Cogn. 15, 285–294 (2006). 16157489 10.1016/j.concog.2005.06.007

[80] K. Kilteni, C. Houborg, H. H. Ehrsson, Rapid learning and unlearning of predicted sensory delays in self-generated touch. eLife 8, e42888 (2019). 31738161 10.7554/eLife.42888 PMC6860990

[81] J. G. Kepecs, M. Robin, C. Munro, Tickle: The organization of a patterned response. Arch. Gen. Psychiatry 5, 237–245 (1961). 13752575 10.1001/archpsyc.1961.01710150019004

[82] T. Kishi, T. Nozawa, A. Nibori, H. Futaki, Y. Miura, M. Shina, K. Matsuki, H. Yanagino, S. Cosentino, K. Hashimoto, A. Takanishi, “One DoF robotic hand that makes human laugh by tickling through rubbing underarm,” in *2016 IEEE International Conference on Intelligent Robots and Systems (IROS)* (IEEE, 2016), pp. 404–409.

[83] E. Wattendorf, B. Westermann, M. Lotze, K. Fiedler, M. R. Celio, Insular cortex activity and the evocation of laughter. J. Comp. Neurol. 524, 1608–1615 (2016). 26287648 10.1002/cne.23884

[84] D. P. Szameitat, A. J. Szameitat, D. Wildgruber, Vocal expression of affective states in spontaneous laughter reveals the bright and the dark side of laughter. Sci. Rep. 12, 5613 (2022). 35379847 10.1038/s41598-022-09416-1 PMC8980048

[85] B. Westermann, M. Lotze, L. Varra, N. Versteeg, M. Domin, L. Nicolet, M. Obrist, K. Klepzig, L. Marbot, L. Lämmler, K. Fiedler, E. Wattendorf, When laughter arrests speech: fMRI-based evidence. Philos. Trans. R. Soc. B Biol. Sci. 377, 20210182 (2022). 10.1098/rstb.2021.0182 PMC9489293 36126674

[86] C. Triscoli, H. Olausson, U. Sailer, H. Ignell, I. Croy, CT-optimized skin stroking delivered by hand or robot is comparable. Front. Behav. Neurosci. 7, 208 (2013). 24391564 10.3389/fnbeh.2013.00208 PMC3866892

[87] Å. B. Vallbo, Microneurography: How it started and how it works. J. Neurophysiol. 120, 1415–1427 (2018). 29924706 10.1152/jn.00933.2017

[88] N. Katic, R. K. Siqueira, L. Cleland, N. Strzalkowski, L. Bent, S. Raspopovic, H. Saal, Modeling foot sole cutaneous afferents: FootSim. iScience 26, 10584 (2023). 10.1016/j.isci.2022.105874 PMC9829801 36636355

[89] R. Ackerley, R. H. Watkins, “Microneurography: Recordings from single neurons in human peripheral nerves,” in *Somatosensory Research Methods*, N. P. Holmes, Ed. (Springer, 2023), pp. 305–331.

[90] H. Olausson, J. Cole, K. Rylander, F. McGlone, Y. Lamarre, B. G. Wallin, H. Krämer, J. Wessberg, M. Elam, M. C. Bushnell, Å. Vallbo, Functional role of unmyelinated tactile afferents in human hairy skin: Sympathetic response and perceptual localization. Exp. Brain Res. 184, 135–140 (2008). 17962926 10.1007/s00221-007-1175-x

[91] I. Morrison, L. S. Löken, J. Minde, J. Wessberg, I. Perini, I. Nennesmo, H. Olausson, Reduced C-afferent fibre density affects perceived pleasantness and empathy for touch. Brain 134, 1116–1126 (2011). 21378097 10.1093/brain/awr011

[92] S. Dagher, S. Ishiyama, Tickle fetishism: Pleasure beyond playfulness. Front. Psychol. 15, 1342342 (2024). 38633879 10.3389/fpsyg.2024.1342342 PMC11021705

[93] D. H. Tuke, *A Dictionary of Psychological Medicine: Giving the Definition, Etymology and Synonyms of the Terms Used in Medical Psychology with the Symptoms, Treatment, and Pathology of Insanity and the Law of Lunacy in Great Britain and Ireland* (J. & A. Churchill, 1892), vol. 2.

[94] F. Mancini, A. Bauleo, J. Cole, F. Lui, C. A. Porro, P. Haggard, G. D. Iannetti, Whole-body mapping of spatial acuity for pain and touch. Ann. Neurol. 75, 917–924 (2014). 24816757 10.1002/ana.24179 PMC4143958

[95] G. Corniani, H. P. Saal, Tactile innervation densities across the whole body. J. Neurophysiol. 124, 1229–1240 (2020). 32965159 10.1152/jn.00313.2020

[96] Y. Lee, K. Hwang, Skin thickness of Korean adults. Surg. Radiol. Anat. 24, 183–189 (2002). 12375070 10.1007/s00276-002-0034-5

[97] L. Maister, A. Fotopoulou, O. Turnbull, M. Tsakiris, The erogenous mirror: Intersubjective and multisensory maps of sexual arousal in men and women. Arch. Sex. Behav. 49, 2919–2933 (2020). 32533518 10.1007/s10508-020-01756-1 PMC7641941

[98] H. Heger, *The Men with the Pink Triangle: The True, Life-and-Death Story of Homosexuals in the Nazi Death Camps* (Haymarket Books, 2010).

[99] D. P. Szameitat, K. Alter, A. J. Szameitat, C. J. Darwin, D. Wildgruber, S. Dietrich, A. Sterr, Differentiation of emotions in laughter at the behavioral level. Emotion 9, 397–405 (2009). 19485617 10.1037/a0015692

[100] K. Alter, D. Wildgruber, “Laughing out loud! Investigations on different types of laughter,” in *The Oxford Handbook of Voice Perception*, S. Frühholz, P. Belin, Eds. (Oxford Library of Psychology, 2018).

[101] D. P. Szameitat, K. Alter, A. J. Szameitat, D. Wildgruber, A. Sterr, C. J. Darwin, Acoustic profiles of distinct emotional expressions in laughter. J. Acoust. Soc. Am. 126, 354–366 (2009). 19603892 10.1121/1.3139899

[102] D. P. Szameitat, B. Kreifelts, K. Alter, A. J. Szameitat, A. Sterr, W. Grodd, D. Wildgruber, It is not always tickling: Distinct cerebral responses during perception of different laughter types. Neuroimage 53, 1264–1271 (2010). 20600991 10.1016/j.neuroimage.2010.06.028

[103] D. Wildgruber, D. P. Szameitat, T. Ethofer, C. Brück, K. Alter, W. Grodd, B. Kreifelts, Different types of laughter modulate connectivity within distinct parts of the laughter perception network. PLOS ONE 8, e63441 (2013). 23667619 10.1371/journal.pone.0063441 PMC3648477

[104] C. Darwin, *The Expression of the Emotions in Man and Animals* (Oxford Univ. Press, UK, 2009); https://isbnsearch.org/isbn/0195392280.

[105] S.-J. Blakemore, D. Wolpert, C. Frith, Why can’t you tickle yourself? Neuroreport 11, R11–R16 (2000). 10943682 10.1097/00001756-200008030-00002

[106] D. A. Leavens, K. A. Bard, Tickling. Curr. Biol. 26, R91–R93 (2016). 26859273 10.1016/j.cub.2015.06.014

[107] M. Haruno, D. M. Wolpert, M. Kawato, MOSAIC model for sensorimotor learning and control. Neural Comput. 13, 2201–2220 (2001). 11570996 10.1162/089976601750541778

[108] D. M. Wolpert, Z. Ghahramani, Computational principles of movement neuroscience. Nat. Neurosci. 3, 1212–1217 (2000). 11127840 10.1038/81497

[109] S. S. Shergill, P. M. Bays, C. D. Frith, D. M. Wolpert, Two eyes for an eye: The neuroscience of force escalation. Science 301, 187 (2003). 12855800 10.1126/science.1085327

[110] P. M. Bays, D. M. Wolpert, J. R. Flanagan, Perception of the consequences of self-action is temporally tuned and event driven. Curr. Biol. 15, 1125–1128 (2005). 15964278 10.1016/j.cub.2005.05.023

[111] P. M. Bays, J. R. Flanagan, D. M. Wolpert, Attenuation of self-generated tactile sensations is predictive, not postdictive. PLOS Biol. 4, e28 (2006). 16402860 10.1371/journal.pbio.0040028 PMC1334241

[112] K. Kilteni, H. H. Ehrsson, Body ownership determines the attenuation of self-generated tactile sensations. Proc. Natl. Acad. Sci. U.S.A. 114, 8426–8431 (2017). 28716932 10.1073/pnas.1703347114 PMC5547616

[113] K. Kilteni, H. H. Ehrsson, Sensorimotor predictions and tool use: Hand-held tools attenuate self-touch. Cognition 165, 1–9 (2017). 28458089 10.1016/j.cognition.2017.04.005

[114] K. Kilteni, B. J. Andersson, C. Houborg, H. H. Ehrsson, Motor imagery involves predicting the sensory consequences of the imagined movement. Nat. Commun. 9, 1617 (2018). 29691389 10.1038/s41467-018-03989-0 PMC5915435

[115] K. Kilteni, P. Engeler, H. H. Ehrsson, Efference copy is necessary for the attenuation of self-generated touch. iScience 23, 100843 (2020). 32058957 10.1016/j.isci.2020.100843 PMC6997587

[116] K. Kilteni, P. Engeler, I. Boberg, L. Maurex, H. H. Ehrsson, No evidence for somatosensory attenuation during action observation of self-touch. Eur. J. Neurosci. 54, 6422–6444 (2021). 34463971 10.1111/ejn.15436

[117] K. Kilteni, H. H. Ehrsson, Predictive attenuation of touch and tactile gating are distinct perceptual phenomena. iScience 25, 104077 (2022). 35372807 10.1016/j.isci.2022.104077 PMC8968059

[118] X. Job, K. Kilteni, Action does not enhance but attenuates predicted touch. eLife 12, e90912 (2023). 38099521 10.7554/eLife.90912 PMC10723797

[119] L. Timar, X. Job, J.-J. Orban de Xivry, K. Kilteni, Aging exerts a limited influence on the perception of self-generated and externally generated touch. J. Neurophysiol. 130, 871–882 (2023). 37609705 10.1152/jn.00145.2023 PMC10642979

[120] N. Wolpe, J. N. Ingram, K. A. Tsvetanov, L. Geerligs, R. A. Kievit, R. N. Henson, D. M. Wolpert, Cam-CAN, J. B. Rowe, Ageing increases reliance on sensorimotor prediction through structural and functional differences in frontostriatal circuits. Nat. Commun. 7, 13034 (2016). 27694879 10.1038/ncomms13034 PMC5063954

[121] M. Parthasharathy, D. Mantini, J.-J. Orban de Xivry, Increased upper-limb sensory attenuation with age. J. Neurophysiol. 127, 474–492 (2022). 34936521 10.1152/jn.00558.2020

[122] N. Valé, I. Tomić, Z. Gironés, D. M. Wolpert, K. Kilteni, P. Bays, Divisive attenuation based on noisy sensorimotor predictions accounts for excess variability in self-touch. bioRxiv 599826 [Preprint] (2024). 10.1101/2024.06.20.599826. PMC7617943 40531356

[123] N. Cemeljic, X. Job, K. Kilteni, Predictions of bimanual self-touch determine the temporal tuning of somatosensory perception. iScience 28, 111643 (2025). 39898028 10.1016/j.isci.2024.111643 PMC11787602

[124] E. Asimakidou, X. Job, K. Kilteni, The positive dimension of schizotypy is associated with a reduced attenuation and precision of self-generated touch. Schizophrenia 8, 57 (2022). 35854009 10.1038/s41537-022-00264-6 PMC9261081

[125] M. D. Hesse, N. Nishitani, G. R. Fink, V. Jousmäki, R. Hari, Attenuation of somatosensory responses to self-produced tactile stimulation. Cereb. Cortex 20, 425–432 (2010). 19505992 10.1093/cercor/bhp110

[126] K. Kilteni, H. H. Ehrsson, Functional connectivity between the cerebellum and somatosensory areas implements the attenuation of self-generated touch. J. Neurosci. 40, 894–906 (2020). 31811029 10.1523/JNEUROSCI.1732-19.2019 PMC6975290

[127] T. B. Crapse, M. A. Sommer, Corollary discharge across the animal kingdom. Nat. Rev. Neurosci. 9, 587–600 (2008). 18641666 10.1038/nrn2457 PMC5153363

[128] K. E. Cullen, Sensory signals during active versus passive movement. Curr. Opin. Neurobiol. 14, 698–706 (2004). 15582371 10.1016/j.conb.2004.10.002

[129] J. F. A. Poulet, B. Hedwig, Corollary discharge inhibition of ascending auditory neurons in the stridulating cricket. J. Neurosci. 23, 4717–4725 (2003). 12805311 10.1523/JNEUROSCI.23-11-04717.2003 PMC6740803

[130] K. Gupta, R. R. Chowdhury, S. Chakrabarti, C. Schwarz, Discerning state estimation and sensory gating, two presumptive predictive signals in mouse barrel cortex. bioRxiv 573180 [Preprint] (2023). 10.1101/2023.12.23.573180.

[131] T. Ishikawa, S. Tomatsu, J. Izawa, S. Kakei, The cerebro-cerebellum: Could it be loci of forward models? Neurosci. Res. 104, 72–79 (2016). 26704591 10.1016/j.neures.2015.12.003

[132] A. A. Sokolov, R. C. Miall, R. B. Ivry, The cerebellum: Adaptive prediction for movement and cognition. Trends Cogn. Sci. 21, 313–332 (2017). 28385461 10.1016/j.tics.2017.02.005 PMC5477675

[133] D. M. Wolpert, R. C. Miall, M. Kawato, Internal models in the cerebellum. Trends Cogn. Sci. 2, 338–347 (1998). 21227230 10.1016/s1364-6613(98)01221-2

[134] K. Kilteni, C. Houborg, H. H. Ehrsson, Brief temporal perturbations in somatosensory reafference disrupt perceptual and neural attenuation and increase supplementary motor area–cerebellar connectivity. J. Neurosci. 43, 5251–5263 (2023). 37339879 10.1523/JNEUROSCI.1743-22.2023 PMC10342225

[135] K. Kilteni, H. H. Ehrsson, Dynamic changes in somatosensory and cerebellar activity mediate temporal recalibration of self-touch. Commun. Biol. 7, 522 (2024). 38702520 10.1038/s42003-024-06188-4 PMC11068753

[136] C. Hull, Prediction signals in the cerebellum: Beyond supervised motor learning. eLife 9, e54073 (2020). 32223891 10.7554/eLife.54073 PMC7105376

[137] S. S. Shergill, T. P. White, D. W. Joyce, P. M. Bays, D. M. Wolpert, C. D. Frith, Modulation of somatosensory processing by action. Neuroimage 70, 356–362 (2013). 23277112 10.1016/j.neuroimage.2012.12.043 PMC4157453

[138] H. Frenzel, J. Bohlender, K. Pinsker, B. Wohlleben, J. Tank, S. G. Lechner, D. Schiska, T. Jaijo, F. Rüschendorf, K. Saar, J. Jordan, J. M. Millán, M. Gross, G. R. Lewin, A genetic basis for mechanosensory traits in humans. PLOS Biol. 10, e1001318 (2012). 22563300 10.1371/journal.pbio.1001318 PMC3341339

[139] V. E. Abraira, D. D. Ginty, The sensory neurons of touch. Neuron 79, 618–639 (2013). 23972592 10.1016/j.neuron.2013.07.051 PMC3811145

[140] B. Pleger, A. Villringer, The human somatosensory system: From perception to decision making. Prog. Neurobiol. 103, 76–97 (2013). 23123624 10.1016/j.pneurobio.2012.10.002

[141] C. Mccabe, E. T. Rolls, A. Bilderbeck, F. McGlone, Cognitive influences on the affective representation of touch and the sight of touch in the human brain. Soc. Cogn. Affect. Neurosci. 3, 97–108 (2008). 19015100 10.1093/scan/nsn005 PMC2555465

[142] C. Krahé, M. von Mohr, A. Gentsch, L. Guy, C. Vari, T. Nolte, A. Fotopoulou, Sensitivity to CT-optimal, affective touch depends on adult attachment style. Sci. Rep. 8, 14544 (2018). 30266979 10.1038/s41598-018-32865-6 PMC6162325

[143] S. Dorros, A. Hanzal, C. Segrin, The Big Five personality traits and perceptions of touch to intimate and nonintimate body regions. J. Res. Pers. 42, 1067–1073 (2008).

[144] R. M. Peters, E. Hackeman, D. Goldreich, Diminutive digits discern delicate details: Fingertip size and the sex difference in tactile spatial acuity. J. Neurosci. 29, 15756–15761 (2009). 20016091 10.1523/JNEUROSCI.3684-09.2009 PMC3849661

[145] R. M. Peters, D. Goldreich, Tactile spatial acuity in childhood: Effects of age and fingertip size. PLOS ONE 8, e84650 (2013). 24454612 10.1371/journal.pone.0084650 PMC3891499

[146] Y. K. Dillon, J. Haynes, M. Henneberg, The relationship of the number of Meissner’s corpuscles to dermatoglyphic characters and finger size. J. Anat. 199, 577–584 (2001). 11760888 10.1046/j.1469-7580.2001.19950577.x PMC1468368

[147] B. Li, G. J. Gerling, B. Li, G. J. Gerling, An individual’s skin stiffness predicts their tactile discrimination of compliance. J. Physiol. 601, 5777–5794 (2023). 37942821 10.1113/JP285271 PMC10872733

[148] L. M. Haag, S. Heba, M. Lenz, B. Glaubitz, O. Höffken, T. Kalisch, N. A. Puts, R. A. E. Edden, M. Tegenthoff, H. Dinse, T. Schmidt-Wilcke, Resting BOLD fluctuations in the primary somatosensory cortex correlate with tactile acuity. Cortex 64, 20–28 (2015). 25461704 10.1016/j.cortex.2014.09.018 PMC5527676

[149] S. Yoshimura, W. Sato, T. Kochiyama, S. Uono, R. Sawada, Y. Kubota, M. Toichi, Gray matter volumes of early sensory regions are associated with individual differences in sensory processing. Hum. Brain Mapp. 38, 6206–6217 (2017). 28940867 10.1002/hbm.23822 PMC6867006

[150] H. Holle, K. Warne, A. K. Seth, H. D. Critchley, J. Ward, Neural basis of contagious itch and why some people are more prone to it. Proc. Natl. Acad. Sci. U.S.A. 109, 19816–19821 (2012). 23150550 10.1073/pnas.1216160109 PMC3511754

[151] M. Schaefer, H.-J. Heinze, M. Rotte, Touch and personality: Extraversion predicts somatosensory brain response. Neuroimage 62, 432–438 (2012). 22584236 10.1016/j.neuroimage.2012.05.004

[152] S. T. Selden, Tickle. J. Am. Acad. Dermatol. 50, 93–97 (2004). 14699372 10.1016/s0190-9622(03)02737-3

[153] V. Juárez-Ramos, E. Salazar-López, M. Á. R. Artacho, K. Chmielowiec, A. Riquelme, J. Fernández-Gómez, A. I. Fernández-Ramirez, A. Vicente de Haro, A. Miranda, M. Caballero, B. Machado, A. G. Hernández, E. G. Milán, The laughter of ticklishness is a Darwinian feature related to empathy in both genders: Self-esteem in men and sexism in women. Open J. Med. Psychol. 3, 18–23 (2014).

[154] R. R. Provine, *Laughter: A Scientific Investigation* (Penguin, 2001).

